# Postural stability impairments in non-athletic individuals after anterior cruciate ligament reconstruction: a cross-sectional study

**DOI:** 10.1186/s13102-026-01543-w

**Published:** 2026-01-20

**Authors:** Gürkan Demi̇rtaş, Hilal Yağar, Emin Kürşat Bulut, Kadir Eren Bi̇çer

**Affiliations:** 1 Department of Physiotherapy and Rehabilitation, Faculty of Bor Health Sciences, Niğde Ömer Halisdemir University, Niğde, Türkiye; 2 Department of Orthopedics and Traumatology, Faculty of Medicine, Niğde Ömer Halisdemir University, Niğde, Türkiye

**Keywords:** Knee joint, Balance, Sedentary behavior, Proprioception, Recovery of function

## Abstract

**Background:**

Anterior cruciate ligament (ACL) injuries are common knee injuries that impair postural stability due to the loss of mechanoreceptors, affecting proprioceptive feedback and sensorimotor control. The aim of our study was to comprehensively investigate the postural stability in non-athletic individuals who had undergone ACL reconstruction (ACLR) and exploring their relationship with patient-reported outcomes.

**Methods:**

A total of 58 participants were included in the study, consisting of 29 individuals who underwent ACLR and 29 control group participants matched for age, height, weight, and sex. Static stability (bipodal and single-leg) was assessed using a force plate by evaluating center of pressure (COP) parameters, while dynamic stability was measured with the Y-Balance Test. During tests, the operated limb in the ACL group was compared with the non-dominant lower extremity of the control group. Group comparisons were performed using independent sample t-tests and Mann–Whitney U tests, and within-group analyses were conducted using paired sample t-tests or Wilcoxon tests. Spearman correlation analysis was used, and statistical significance was set at *p* < 0.05.

**Results:**

When comparing bipodal stance scores between the groups, the ACL group exhibited greater anteroposterior amplitude of the COP sway (*p* < 0.001). No differences were observed between the operated and non-operated limbs in single-leg stance parameters (*p* > 0.05); however, when compared with the control group’s non-dominant limb, the control values were better than the operated limb (*p* < 0.05). In the Y-Balance Test, anterior reach and composite scores were significantly lower on the operated limb (*p* < 0.05). When compared with the control group, the ACL group demonstrated lower performance in anterior, posterolateral and posteromedial reaches, as well as composite scores (*p* < 0.05). No clinically relevant limb-symmetry deficits were observed within the ACL group (*p* > 0.05).

**Conclusion:**

In non-athletic individuals, limb symmetry was achieved in static postural stability tests post-operatively; however, differences compared to healthy individuals persisted. Postural deficits were more pronounced particularly in single-leg stance and dynamic postural stability tasks. These findings suggest that postural stability deficits in non-athletic populations may persist at least 12 months post-surgery.

**Trial registration:**

NCT07210853 retrospectively registered. ( date29/09/2025).

## Introduction

Anterior cruciate ligament (ACL) injuries are among the most common knee injuries, particularly in athletes, leading to functional impairments and motor coordination deficits [1, 2]. Postural stability requires the sensory perception of body movements, the integration of sensorimotor information within the central nervous system, and the generation of an appropriate motor response [3]. The functional stability of the knee joint, particularly during voluntary movements, is primarily regulated by the sensorimotor system. This dynamic system integrates proprioceptive, vestibular, and visual information within the central nervous system, enabling adaptation to environmental demands [1].

The ACL mechanically restricts excessive joint movements and plays an important role in proprioceptive feedback through the mechanoreceptors present within its structure [4]. Proprioception, a component of the somatosensory system that provides information about joint position, movement, and posture, is regulated by mechanoreceptors such as Ruffini endings, Pacinian corpuscles, and free nerve endings within the ACL [5]. Damage to these mechanoreceptors following ACL rupture disrupts the afferent feedback mechanism, leading to irregular stabilization reflexes and negatively affecting postural stability [6].

ACL reconstruction (ACLR) is performed to restore the mechanical stability of the knee joint and facilitate the return to functional activities [4]. Previous studies have demonstrated that ACLR largely restores knee stability and function. However, findings regarding the complete restoration of sensory function remain inconsistent [4, 7–9]. Since the proprioceptive receptors of the ACL are part of the reflex arc responsible for stable muscle contractions, damage to this structure reduces the sensory input transmitted to the nervous system, significantly impairing the knee’s stabilization capacity [10]. The impairment of this feedback mechanism after ACL injury directly affects postural control strategies [4]. This condition leads to a systematic alteration in the sensorimotor control system, potentially resulting in persistent functional deficits following ACLR [9, 11].

Postural stability has been reported to influence reinjury risk, the biomechanics of daily living activities such as walking and running, and neuromuscular control following ACL reconstruction. Therefore, it is considered an important clinical indicator for assessing sensorimotor alterations after ACL reconstruction [12]. Postural stability is commonly assessed by force plate measurements that analyze center of pressure (COP) displacement, while computerized posturography and functional balance tests (e.g., single-leg stance) are also used to evaluate postural stability under varying conditions [13–15]. Studies in the literature suggest that different postural stability assessment methods are not directly correlated with each other [16–18]. These studies highlight that various test protocols, measurement principles, and evaluation criteria approaches stability performance from different perspectives. Therefore, multiple assessment tools should be used to comprehensively analyze individuals’ postural stability abilities rather than relying on a single method.

Non-athletic individuals may be less motivated to engage in high-intensity exercises or activities that facilitate recovery, which could influence postoperative outcomes. Moreover, previous research has shown that athletic populations demonstrate superior postural stability and distinct neuromuscular characteristics compared with sedentary individuals. Therefore, postoperative recovery profiles following knee surgery may differ between athletic and non-athletic groups [19, 20]. However, there is insufficient data on postural stability deficits in sedentary individuals or non-athletic populations. Accordingly, our study aims to contribute to the literature by comprehensively examining postural stability parameters in non-athletic individuals who have undergone ACLR and exploring their relationship with patient-reported outcomes.

## Materials and methods

### Study design and participants

This cross-sectional study was conducted at Nigde Ömer Halisdemir University Training and Research Hospital following the approval of Nigde Ömer Halisdemir University Non-Interventional Clinical Research Ethics Committee (register number: 2023/107). The required sample size, calculated using G*Power (B = 0.85, α = 0.05, effect size d = 0.84), was determined to be *n* = 27 participants per group [21]. This study was registered retrospectively for clinical trials with the number NCT07210853 (date:29/09/2025).

Participants were provided with detailed information regarding the study, and written informed consent was obtained from those who voluntarily agreed to participate. Subsequently, a pre-assessment was conducted to evaluate participants based on predetermined inclusion and exclusion criteria. Individuals who met the inclusion criteria and agreed to participate were assessed for static postural stability with force-plate and dynamic postural stability with Y-balance test. In the ACLR group, the operated limb was compared both with the non-operated limb and with the non-dominant limb of the control group [22]. Assessments were performed in a standardized order: static stability (bipodal and single-leg, respectively), dynamic stability using the Y-Balance Test, and finally patient-reported outcome measures. To avoid any learning effect, patients were asked to perform one familiarization trial with both lower limbs. In all tests, the non-operated limb was evaluated first in the ACLR group, whereas the dominant limb was evaluated first in the control group. All assessment were completed within a single session. To minimize potential measurement variability, all assessments were conducted by a physiotherapist with five years of professional experience in the field.

Participants aged 18–40 years who had undergone unilateral ACLR were 12–24 months postoperative, and a Tegner activity score of ≥ 5 was included in the study. Participants were excluded if they had any other orthopedic conditions affecting the lower extremities besides from anterior cruciate ligament injury, a history of lower extremity surgery for any reason, lower limb malalignment, or any neurological or circulatory disorders that could cause postural stability impairments. Additionally, those with conditions affecting vision or the vestibular pathways, a history of orthopedic surgery, a diagnosis of rheumatologic disease, or pregnancy were also excluded. The control group consisted of non-athlete voluntary hospital staff who were matched to the ACL group for age, sex, height, and weight. During control group selection, participants were required to be between 18 and 40 years of age, and the same exclusion criteria as those applied to the ACL group were used. Participants’ dominant foot was identified as the leg used to kick a ball.

### Assessment tools

#### Descriptive information form

A patient evaluation form prepared by the researchers and administered through face-to-face interviews was used to collect demographic and health-related information, including age (years), height (cm), body weight (kg), body mass index (BMI, kg/m²), dominant limb (right/left), history of sports participation, and engagement in regular physical activity.

#### Patient-reported outcome measures (PROM’s)

Patient-reported outcome measures included the Tegner Activity Scale [23], the Knee Injury and Osteoarthritis Outcome Score (KOOS) [24], and the International Knee Documentation Committee (IKDC) subjective form [25]. KOOS and IKDC are validated and commonly used PROMs for assessing patient-reported symptoms, functional limitations, and knee-related quality of life following ACL injury and reconstruction, whereas the Tegner Activity Scale was used to evaluate the participants’ activity levels. Tegner, KOOS, and IKDC were included to provide a more comprehensive understanding of the overall knee condition of the participants.

#### Assessment of static postural stability

Postural stability was evaluated in both bipodal and single-leg standing positions using a force plate (K-plate, Kinvent^®^, Montpellier, France). Tests were performed barefoot, and participants were instructed to place their hands on their waists during the assessment. Each measurement was repeated three times, and the average scores determined by the software to which the force plate was connected were used for the analysis. Before each test, participants were given one practice trial to familiarize themselves with the procedure. The test was repeated if a participant failed to maintain the required posture or removed their hands from their waist.

Center of pressure based stabilometric measurements have been identified as highly sensitive methods for the assessment of postural control, providing reliable indicators of both sensory integration and motor output [13]. Therefore, in the present study we employed variables representing the characteristics of COP displacements in the anteroposterior (AP) and mediolateral (ML) Amplitude (mm), Path Length (mm), and Mean Velocity (mm/s), as well as Ellipse Area (mm^2^), COP Path Length (mm) and Velocity (mm/s) values as global indicators of overall postural stability.

Postural stability parameters were calculated according to the procedures described in the Kinvent Bipodal Balance: Procedures & Testing e-book. Ellipse area was calculated using the covariance method. COP path length was calculated by summing the distances between each consecutive COP point measured during the postural stability task. COP mean velocity was calculated by dividing the total COP path length by the duration of the trial. Amplitude in the AP and ML directions was calculated as the difference between the maximum and minimum COP displacement values along each respective axis. A detailed description of the calculation procedures and mathematical formulas is provided in the Kinvent e-book [26].

During the bipodal stance test, participants were instructed to stand as still as possible for 30 s while focusing on a fixed point at eye level approximately 2 m away on a wall. Bipodal stance was assessed using two independent force plates that were interconnected, allowing separate analysis of the right and left foot in addition to total COP variables. In the single-leg test, participants were required to maintain the tested limb in an extended position while flexing the non-tested limb’s knee to 90° and keeping the hip in a neutral position. They were asked to remain as still as possible for 15 s while focusing on a fixed point at eye level approximately 2 m away. In the literature, single-leg stance tests are typically performed for durations ranging from 10 to 60 s [27]. In the present study, a duration of 15 s was selected because it is considered both clinically feasible and practical to implement.

#### Assessment of dynamic postural stability

##### Y Balance Test

The Y-Balance Test is a simplified and reliable version of the Star Excursion Balance Test (SEBT), incorporating only the three most reliable reach directions (anterior, posterolateral, and posteromedial) to enhance its validity and field applicability. The test was conducted according to previously established protocols [28]. The stance limb was defined as the test limb, and the opposite limb was used for reaching. Reaching distances were visually observed and noted by the researcher. Limb length was determined by measuring the distance between anterosuperior iliac spine and ipsilateral medial malleolus and normalised values were obtained with the formula (reach distance/limb length) * 100. The composite (CP) score was determined using the following formula: [(Anterior reach (cm) + posterolateral reach (cm) + posteromedial reach (cm))/(limb length *3)] * 100) [29].

### Statistical analysis

Statistical analyses were performed using IBM SPSS Statistics 26. Descriptive statistics and frequency tables were used for data interpretation. Parametric methods were applied for the normal distribution of demographic and Y-balance test data. In contrast, non-parametric methods were used for static stability data that did not exhibit normal distribution. For parametric data, independent sample t-tests were used to compare measurements between two independent groups, and paired sample t-tests were used for within-group comparisons. For non-parametric data, the Mann-Whitney U test was used for between-group comparisons, while the Wilcoxon test was used for within-group comparisons. The correlation between test methods was analyzed using Spearman’s correlation test. The limb symmetry index was calculated as (operated limb score / non-operated limb score) × 100 for the ACLR group, and as (non-dominant limb score / dominant limb score) × 100 for the control group. A *p-value* < 0.05 indicated a statistically significant difference.

## Results

A total of 58 participants were included in the study, with 29 individuals in the ACL group and 29 in the control group. Each group consisted of 3 females and 26 males. All participants had no prior sports background and did not exercise regularly during the study. In the ACL group, 20 participants underwent isolated ACLR, and 9 underwent ACLR + meniscus repair. All ACLR surgeries were performed using hamstring tendon autografts. The average postoperative assessment period for the ACL group was 17.45 ± 5.09 months. Additionally, 12 participants completed a postoperative rehabilitation program, receiving an average of 21.67 ± 5.77 sessions. There were no significant differences between the groups regarding age, height, body weight, or BMI (*p* > 0.05). The KOOS scores of the ACL group were 73.28 ± 14.80, the IKDC scores were 59.52 ± 14.12 and the Tegner scores were 5.48 ± 0.78 (Table 1).

**Table 1:** Demographic and clinical characteristics of the participants

	ACL (*n* = 29) Mean ± SD	Control (*n* = 29) Mean ± SD	*p*
Age (years)	28.79 ± 7.87	26.41 ± 5.82	0.196
Height (cm)	173.69 ± 8.39	173.93 ± 6.48	0.903
Weight (kg)	79.93 ± 12.30	78.86 ± 13.41	0.751
BMI (kg/m²)	26.45 ± 3.33	25.98 ± 3.65	0.613
Tegner Activity Score	5.48 ± 0.78	5.86 ± 0.74	0.064
KOOS	73.28 ± 14.80		
IKDC	59.52 ± 14.12		
Postoperative assessment (months)	17.45 ± 5.09		
Rehabilitation sessions (number)	21.67 ± 5.77		
	*n* (%)	*n* (%)	
Gender (Male/Female)	26/3 (89.7/10.3)	26/3 (89.7/10.3)	1.000
Dominant extremity (Right/Left)	25/4 (86.2/13.8)	25/4 (86.2/13.8)	1.000
Operated extremity (Right/Left)	14/15 (48.3/51.7)		
Type of surgery	20^a^/9^b^		
Rehabilitation (Yes/No)	12/17 (41.4/58.6)		

*BMI* body mass index, *KOOS* Knee Injury and Osteoarthritis Outcome Score, *IKDC* International Knee Documentation Committee Subjective Knee Form

^a^Isolated ACL intervention

^b^ACL + meniscus intervention

In the bipodal stability test, no significant differences in stability parameters were observed between the limbs in either group (*p* > 0.05). Similarly, when the operated limb of the ACL group was compared to the non-dominant limb of the control group, no significant differences were found (*p* > 0.05). However, when comparing total scores between the two groups, the AP amplitude value was significantly higher in the ACL group (*p* < 0.001) (Table 2).

**Table 2:** Bipodal postural stability test results of the participants

	ACL (*n* = 29)				Control (*n* = 29)				Operated vs. non-dominant	ACL vs. Control LSI
	Operated Mean ± SD	Non-operated Mean ± SD	*p*	LSI	Dominant Mean ± SD	Non-dominant Mean ± SD	*p*	LSI	*p*	*p*
Ellipse Area (mm^2^)	21.38 ± 23.12	27.17 ± 23.97	0.117	94.62 ± 47.88	18.34 ± 11.17	16.82 ± 17.07	0.294	106.49 ± 75.08	0.118	0.476
AP Amplitude (mm)	19.48 ± 8.32	22.39 ± 8.10	0.247	90.52 ± 28.60	15.94 ± 5.07	16.11 ± 5.56	0.737	106.29 ± 34.74	0.055	0.064
AP Path Length (mm)	216.31 ± 72.98	238.34 ± 53.83	0.119	91.40 ± 25.82	224.80 ± 61.10	222.01 ± 60.43	0.689	102.04 ± 23.55	0.680	0.107
AP Mean Velocity (mm/s)	7.21 ± 2.43	7.95 ± 1.82	0.122	91.36 ± 25.80	7.40 ± 2.02	9.81 ± 3.26	0.567	101.29 ± 24.16	0.581	0.182
ML Amplitude (mm)	4.41 ± 2.73	4.84 ± 3.47	0.596	104.51 ± 40.46	3.77 ± 2.32	3.74 ± 2.42	0.837	109.34 ± 48.95	0.069	0.352
ML Path Length (mm)	69.17 ± 21.40	67.14 ± 18.21	0.393	103.93 ± 20.07	73.15 ± 18.81	70.61 ± 16.60	0.914	99.29 ± 18.14	0.602	0.359
ML Mean Velocity (mm/s)	2.31 ± 0.71	2.24 ± 0.61	0.424	103.92 ± 20.06	2.44 ± 0.62	2.35 ± 0.55	0.880	99.30 ± 18.14	0.592	0.362
COP Path Length (mm)	236.17 ± 76.04	256.07 ± 55.28	0.136	92.52 ± 23.80	247.17 ± 62.79	243.67 ± 61.78	0.634	101.41 ± 21.47	0.519	0.141
COP Mean Velocity (mm/s)	7.88 ± 2.54	8.54 ± 1.86	0.139	92.55 ± 23.80	8.03 ± 2.28	7.89 ± 2.26	0.721	101.00 ± 21.56	0.883	0.162
**Total**	**ACL (*n*** ** = 29)**				**Control (** ***n*** ** = 29)**				**p**	
Ellipse Area (mm^2^)	95.11 ± 57.92				90.29 ± 33.78				0.774	
COP Path Length (mm)	241.21 ± 36.48				248.91 ± 51.43				0.613	
COP Mean Velocity (mm/s)	8.05 ± 1.23				8.30 ± 1.72				0.652	
AP Amplitude (mm)	18.56 ± 4.54				14.66 ± 3.06				< 0.001*	
ML Amplitude (mm)	8.95 ± 5.36				8.96 ± 2.38				0.169	
AP Path Length (mm)	191.72 ± 26.84				188.05 ± 43.31				0.646	
ML Path Length (mm)	118.24 ± 30.02				125.00 ± 28.23				0.316	
AP Mean Velocity (mm/s)	6.40 ± 0.91				6.25 ± 1.43				0.586	
ML Mean Velocity (mm/s)	4.61 ± 1				4.16 ± 0.95				0.095	

*AP* Anteroposterior, *ML* Mediolateral, *COP* Center of Pressure, *p < 0.05, Mann-Whitney U Test

When the groups were compared, the ACL group demonstrated higher values for ellipse area, ML amplitude, AP path length, ML path length, and COP path length (*p* < 0.05). However, no significant differences were found in AP amplitude, AP mean velocity, ML mean velocity, COP mean velocity, AP position, and ML position (*p* > 0.05) (Table 3).

**Table 3:** Single-leg postural stability test results of the participants

	ACL (*n* = 29)				Control (*n* = 29)				Operated vs. non-dominant	ACL vs. Control LSI
	Operated Mean ± SD	Non-operated Mean ± SD	*p*	LSI	Dominant Mean ± SD	Non-dominant Mean ± SD	*p*	LSI	*p*	*p*
Ellipse Area (mm^2^)	780.86 ± 264.85	762.76 ± 216.45	0.762	104.05 ± 32.69	603.15 ± 216.16	633.66 ± 315.90	0.439	107.26 ± 36.69	0.021**	0,726
AP Amplitude (mm)	38.44 ± 9.29	39.33 ± 10.14	0.897	99.72 ± 23.41	32.63 ± 7.56	35.85 ± 14.30	0.223	109.34 ± 28.52	0.189	0,215
ML Amplitude (mm)	29.51 ± 4.73	31.07 ± 5.08	0.095	95.72 ± 14.33	25.60 ± 6.30	25.48 ± 6.24	0.733	101.54 ± 18.73	0.006**	0,190
AP Path Length (mm)	596.69 ± 237.87	624.14 ± 234.98	0.183	95.59 ± 15.34	454.15 ± 165.06	446.41 ± 165.49	0.236	101.43 ± 30.18	0.014**	0,359
ML Path Length (mm)	587.41 ± 215.42	614.21 ± 233.37	0.406	97.25 ± 14.19	457.98 ± 170.54	42,896 ± 143.33	0.264	98.96 ± 31.06	0.005**	0,789
COP Path Length (mm)	922.45 ± 343.90	961.72 ± 351.22	0.341	96.37 ± 12.79	701.07 ± 243.81	681.00 ± 231.45	0.250	100.74 ± 28.79	0.007**	0,459
AP Mean Velocity (mm/s)	23.98 ± 5.10	25.13 ± 5.22	0.202	95.74 ± 15.22	23.32 ± 6.80	22.85 ± 7.61	0.241	98.87 ± 21.63	0.423	0,527
ML Mean Velocity (mm/s)	23.92 ± 4.93	24.96 ± 5.82	0.439	97.32 ± 14.34	23.41 ± 6.72	22.10 ± 6.51	0.290	96.48 ± 22.71	0.294	0,868
COP Mean Velocity (mm/s)	37.32 ± 7.20	38.91 ± 7.84	0.393	96.49 ± 12.96	36.22 ± 10.19	34.97 ± 10.48	0.183	97.80 ± 20.25	0.327	0,771

*AP* Anteroposterior, *ML* Mediolateral, *COP* Center of Pressure, *p < 0.05, Wilcoxon test. **p < 0.05, Mann-Whitney U Test

In the Y-balance test, the operated limb in the ACL group performed significantly lower in terms of anterior (cm), anterior (%), and composite score (%) compared to the non-operated limb (*p* = 0.001, *p* = 0.001 and *p* = 0.018, respectively). When the operated limb was compared to the non-dominant limb of the control group, the operated limb showed significantly lower values in all Y-Balance Test parameters (Table 4).

**Table 4:** Participants’ Y-balance test results

		ACL (*n* = 34)				Control (*n* = 34)				Operated vs. non-dominant	ACL vs. Control LSI
		Operated Mean ± SD	Non-operated Mean ± SD	*p*	LSI	Dominant Mean ± SD	Non-dominant Mean ± SD	*p*	LSI	*p*	*p*
Anterior	cm	71.00 ± 5.41	73.95 ± 5.77	0.001*	96.17 ± 5.57	76.56 ± 6.75	76.90 ± 6.71	0.564	100.53 ± 4.14	0.001**	0.001**
	(%)	82.24 ± 6.05	85.62 ± 5.97	0.001*		85.53 ± 7.60	85.92 ± 7.61	0.556		0.046**	
Posterolateral	cm	79.04 ± 14.47	80.10 ± 14.99	0.430	99.23 ± 9.85	91.55 ± 8.31	92.83 ± 8.26	0.100	101.51 ± 4.64	0.001**	0.267
	(%)	91.37 ± 15.54	92.63 ± 16.35	0.422		102.28 ± 9.34	101.49 ± 11.94	0.123		0.001**	
Posteromedial	cm	82.55 ± 8.80	84.64 ± 9.82	0.056	97.85 ± 6.53	91.69 ± 8.57	90.81 ± 10.27	0.376	99.00 ± 6.07	0.002**	0.490
	(%)	95.58 ± 9.71	97.96 ± 10.46	0.061		102.46 ± 9.99	101.49 ± 11.94	0.383		0.043**	
Composite score	(%)	89.73 ± 9.04	92.07 ± 9.44	0.018*	97.62 ± 5.47	99.00 ± 6.07	100.29 ± 3.39	0.687	100.29 ± 3.39	0.006**	0.030**

*p < 0.05, Paired Sample-t. **p < 0.05, Independent Sample-t

The limb symmetry index values of ACL patients were > 90 in all tests, indicating no clinically significant differences.

Regarding static stability assessments, bipodal COP measures show non-significant correlations with PROMs. Unilateral COP mean velocity is negatively correlated with IKDC (*r* = -0.354, *p* < 0.01) and Tegner activity scale (*r* = -0.368, *p* < 0.05). In the Y-balance test, posterolateral reach exhibits positive correlations with KOOS (*r* = 0.524, *p* < 0.01), IKDC (*r* = 0.534, *p* < 0.01), and Tegner activity scale (*r* = 0.403, *p* < 0.05). Similarly, medial reach correlates positively with Tegner (*r* = 0.392, *p* < 0.05). The composite Y-Balance score shows significant positive correlations with KOOS (*r* = 0.495, *p* < 0.01), IKDC (*r* = 0.448, *p* < 0.05), and Tegner (*r* = 0.378, *p* < 0.05) (Table 5).

**Table 5:** Correlation of postural stability assessment parameters with patient reported outcome measures

	(1)	(2)	(3)	(4)	(5)	(6)	(7)	(8)	(9)	(10)	(11)	(12)	(13)
(1) KOOS	1												
(2) IKDC	0.831^**^	1											
(3) Tegner Activity Scale	0.520^**^	0.574^**^	1										
(4) Bipodal COP ellipse area	− 0.127	− 0.261	− 0.223	1									
(5) Bipodal COP path length	0.043	− 0.110	− 0.259	0.530^**^	1								
(6) Bipodal COP Mean Velocity	0.045	− 0.098	− 0.244	0.539^**^	0.997^**^	1							
(7) Single-leg (op) COP ellipse area	− 0.115	− 0.271	− 0.291	0.117	0.204	0.201	1						
(8) Single-leg (op) COP path length	− 0.246	− 0.262	− 0.198	0.209	0.224	0.228	0.324	1					
(9) Single-leg (op) COP Mean Velocity	− 0.227	− 0.354^*^	− 0.368^*^	0.024	0.392^*^	0.372^*^	0.433^*^	0.547^**^	1				
(10) YBT-(op) anterior (%)	0.127	0.056	0.060	− 0.164	0.105	0.105	0.318	0.199	0.018	1			
(11) YBT-(op) posterolateral (%)	0.524^**^	0.534^**^	0.403^*^	− 0.189	− 0.075	− 0.079	− 0.129	− 0.476^**^	− 0.348	0.411^*^	1		
(12) YBT-(op) posteromedial (%)	0.264	0.246	0.392^*^	0.019	0.089	0.088	0.052	− 0.077	− 0.229	0.654^**^	0.705^**^	1	
(13) YBT-(op) composite	0.495^**^	0.448^*^	0.378^*^	− 0.133	0.024	0.022	− 0.023	− 0.250	− 0.282	0.659^**^	0.931^**^	0.866^**^	1

*COP* Center of pressure, *Single-leg (op)* single leg test of the operated limb, *YBT-(op)* Y balance test of the operated limb, *p < 0.05, **p < 0.01

## Discussion

Anterior cruciate ligament injuries can significantly affect postural stability due to proprioceptive impairments and loss of sensorimotor control. These impairments may negatively impact individuals’ long-term functional capacity and increase the risk of re-injury. This study investigates postural stability changes in non-athletic individuals 12–24 months after ACL reconstruction and points to the possibility that postural stability impairments may still be present. Additionally, incorporating static and dynamic stability assessments provides a multidimensional perspective and integrates subjective function with objective performance by correlating stability parameters with patient-reported outcomes in non-athletic individuals.

The bipodal stability test is a safe method for assessing postural stability in the early postoperative period. A previous study reported no significant difference between the two groups among participants who had undergone surgery an average of 18 months prior [30]. Similarly, no significant difference was reported between ACLR patients and controls at an average of 36 months post-surgery [31]. Consistent with these findings, our study showed no significant differences in postural stability between the operated, non-operated, and non-dominant limbs of the control group during the bipodal stability test. However, it should be noted that the bipodal stance test may not possess sufficient sensitivity or challenge to detect subtle postural stability impairments that may occur following anterior cruciate ligament reconstruction [32].

The single-leg stability test is one of the most commonly used tests after ACLR and allows for comparisons between the operated and non-operated limbs. Our study observed no significant differences between the operated and non-operated limbs. However, when comparing the operated limb to the non-dominant limb of the control group, significant differences were found in the ellipse area and several anteroposterior and mediolateral stability parameters. Previous studies have reported increased anteroposterior and mediolateral sway in ACLR patients [4, 7]. Similarly, a systematic review indicated that single-leg stability tests postoperatively showed significantly increased sway in both directions, with notable differences between limbs [1].

The Y-Balance Test is a valid and reliable method for assessing dynamic postural stability after ACLR [33]. A study evaluating patients two years post-surgery found no significant differences in Y-balance test scores between limbs or compared to control groups [22]. Another study found no differences between limbs at two to three years postoperatively but reported worse anterior reach performance in the affected limb compared to the control group [21]. Our study observed significantly lower performance in all directions and composite scores for the operated limb compared to the non-dominant limb. We attribute this difference to the fact that our study included participants who were 12–24 months postoperative, during which the recovery process may still be ongoing. Additionally, since our participants were sedentary and did not have athletic performance concerns, they may have avoided demanding activities necessary for recovery after surgery.

Another key finding in our study was that the operated limb performed worse in the anterior direction and composite score compared to the non-operated limb. Previous studies suggest that anterior reach requires greater quadriceps activation, and due to quadriceps-hamstring muscle imbalances, the tibia may not effectively resist anterior translation [22, 34, 35]. Another explanation is that participants may have adopted a more upright posture requiring less hip flexion, which reduced forward pelvic movement and resulted in greater reliance on knee extensors [36]. Studies indicate that a limb difference of > 4 cm in the anterior direction [37], and > 12 cm in the composite score [38, 39] may be risk factors for future injuries [33]. Based on these findings, while the limb differences in our study were statistically significant, they were not clinically significant.

When evaluating the limb symmetry index (LSI), values were found to be > 90% in all three assessment methods (bipodal stance, single-leg stance, and Y-Balance Test). This result suggests that postural stability differences between limbs are not clinically present in individuals more than one-year post-surgery [22]. Previous studies have reported that postural stability declines in the early postoperative period but improves between six months and one year and continues improving for up to two years [4]. Significant improvements in postural stability have been reported up to one year postoperatively, with further enhancements observed for up to two years [40]. These findings are consistent with our results.

Our study found no significant relationship between PROMs and static stability outcomes, whereas a moderate correlation was observed with the dynamic stability test. The findings of our study are consistent with previous research demonstrating significant correlations between Y-Balance Test composite scores and both subjective clinical outcomes and functional performance measures [41, 42]. The Y-balance test is associated with parameters such as knee muscle strength and joint laxity, making it a more effective method for evaluating functional performance after ACLR than static postural stability tests [18, 36]. In this context, and as also reported in the literature, the moderate associations observed between Y-Balance test performance and PROMs such as KOOS, IKDC, and Tegner suggest that dynamic postural stability assessments may be clinically more meaningful in reflecting subjective recovery perceptions following ACLR. Moreover, the absence of a relationship between static and dynamic postural stability parameters in our study further supports this notion.

There is ongoing debate in the literature regarding which limb should be used for comparison in control groups [1]. A previous study suggested that leg dominance should be considered comparing ACLR patients with healthy individuals, as postural deficits become more apparent when compared to the dominant limb [43]. However, other studies have reported that limb dominance does not affect postural stability in healthy individuals [3, 8]. In our study, we chose to compare the ACLR limb to the non-dominant limb of the control group because this approach has commonly been used in previous research [22].

One of the strengths of our study is that, instead of focusing solely on an athletic population, we included participants with no prior sports history. This increases the generalizability of our findings to a broader population; additionally, including a control group allowed for an objective comparison of ACLR individuals with healthy individuals. The use of multiple tests and measurement methods enhanced our assessments’ comprehensiveness, contributing to our results’ reliability.

However, our study has some limitations. A few participants underwent a rehabilitation program and those who did attend programs at different institutions, making it difficult to standardize the rehabilitation content. This raises the question of whether rehabilitation influenced the results. In addition, some participants had meniscal intervention, which reduced the homogeneity of the sample and made interpretation more complex. Lower extremity muscle strength, particularly that of the quadriceps and hamstrings, is crucial in maintaining postural stability. The lack of muscle strength assessment in these muscle groups is another limitation of our study. Future studies should employ longitudinal designs and include additional clinical variables such as muscle strength, pain, and landing mechanics to more comprehensively understand the mechanisms underlying postural stability after ACL reconstruction. Moreover, research involving larger and more homogeneous samples, as well as comparisons between athletic and non-athletic populations, is warranted to clarify population-specific recovery profiles.

## Conclusion

These findings suggest that postural stability impairments in non-athletic individuals following ACL reconstruction may persist not only in the early postoperative period, but also beyond the first year after surgery. Accordingly, incorporating targeted stabilization exercises into the rehabilitation process may enhance long-term functional capacity and contribute to reducing the risk of potential reinjury.

## Data Availability

The data supporting the study’s findings are available from the corresponding author upon reasonable request.

## Abbreviations

ACL: Anterior cruciate ligament
ACLR: ACL reconstruction
PROM’s: Patient-Reported Outcome Measures
KOOS: PROM’s, Knee Injury and Osteoarthritis Outcome Score
IKDC: International Knee Documentation Committee
SEBT: Star Excursion Balance Test
CP: The composite score
AP: Anteroposterior
BMI: Body Mass Index
ML: Mediolateral
COP: Center of Pressure

## References

[1] Lehmann T, Paschen L, Baumeister J. Single-Leg assessment of postural stability after anterior cruciate ligament injury: a systematic review and Meta-Analysis. Sports Med Open. 2017;3. 10.1186/S40798-017-0100-5. 10.1186/s40798-017-0100-5 PMC5574832 28853022

[2] Denti M, Randelli P, Lo Vetere D, Moioli M, Bagnoli I, Cawley PW. Motor control performance in the lower extremity: normals vs. anterior cruciate ligament reconstructed knees 5–8 years from the index surgery. Knee Surg Sports Traumatol Arthrosc. 2000;8:296–300. 10.1007/S001670000136. 11061299 10.1007/s001670000136

[3] Alonso AC, Greve JMDA, Camanho GL. Evaluating the center of gravity of dislocations in soccer players with and without reconstruction of the anterior cruciate ligament using a balance platform. Clin (Sao Paulo). 2009;64:163–70. 10.1590/S1807-59322009000300003. 10.1590/S1807-59322009000300003 PMC2666448 19330239

[4] Brophy RH, Schafer KA, Knapik DM, Motley J, Haas A, Matava MJ, et al. Changes in dynamic postural stability after ACL reconstruction: results over 2 years of Follow-up. Orthop J Sports Med. 2022;10. 10.1177/23259671221098989. 10.1177/23259671221098989 PMC9201321 35722181

[5] Banios K, Raoulis V, Fyllos A, Chytas D, Mitrousias V, Zibis A. Anterior and Posterior Cruciate Ligaments Mechanoreceptors: A Review of Basic Science. Diagnostics 2022, Vol 12, Page 331. 2022;12:331. 10.3390/DIAGNOSTICS12020331. 10.3390/diagnostics12020331 PMC8870829 35204424

[6] Kapreli E, Athanasopoulos S. The anterior cruciate ligament deficiency as a model of brain plasticity. Med Hypotheses. 2006;67:645–50. 10.1016/J.MEHY.2006.01.063. 16698187 10.1016/j.mehy.2006.01.063

[7] Mohammadi F, Salavati M, Akhbari B, Mazaheri M, Khorrami M, Negahban H. Static and dynamic postural control in competitive athletes after anterior cruciate ligament reconstruction and controls. Knee Surgery, sports traumatology. Arthroscopy. 2012;20:1603–10. 10.1007/S00167-011-1806-4/METRICS. 10.1007/s00167-011-1806-4 22124847

[8] Zouita Ben Moussa A, Zouita S, Dziri C, Ben Salah FZ. Single-leg assessment of postural stability and knee functional outcome two years after anterior cruciate ligament reconstruction. Ann Phys Rehabil Med. 2009;52:475–84. 10.1016/J.REHAB.2009.02.006. 19477706 10.1016/j.rehab.2009.02.006

[9] Valeriani M, Restuccia D, Di Lazzaro V, Franceschi F, Fabbriciani C, Tonali P. Clinical and neurophysiological abnormalities before and after reconstruction of the anterior cruciate ligament of the knee. Acta Neurol Scand. 1999;99:303–7. 10.1111/J.1600-0404.1999.TB00680.X. 10348160 10.1111/j.1600-0404.1999.tb00680.x

[10] Güzel N, Genç AS, Yılmaz AK, Kehribar L. The relationship between lower extremity functional performance and balance after anterior cruciate ligament reconstruction: results of patients treated with the modified All-Inside technique. J Pers Med. 2023;13. 10.3390/JPM13030466. 10.3390/jpm13030466 PMC10052949 36983648

[11] Lai CCH, Ardern CL, Feller JA, Webster KE. Eighty-three per cent of elite athletes return to preinjury sport after anterior cruciate ligament reconstruction: a systematic review with meta-analysis of return to sport rates, graft rupture rates and performance outcomes. Br J Sports Med. 2018;52:128–38. 10.1136/BJSPORTS-2016-096836. 28223305 10.1136/bjsports-2016-096836

[12] Sigward SM, Lin P, Pratt K. Knee loading asymmetries during gait and running in early rehabilitation following anterior cruciate ligament reconstruction: A longitudinal study. Clin Biomech Elsevier Ltd. 2016;32:249–54. 10.1016/j.clinbiomech.2015.11.003. 10.1016/j.clinbiomech.2015.11.003 26640045

[13] Paillard T, Noé F. Techniques and methods for testing the postural function in healthy and pathological subjects. Biomed Res Int. 2015;2015:891390. 10.1155/2015/891390. 26640800 10.1155/2015/891390 PMC4659957

[14] Julienne A, Verbecque E, Besnard S. Normative data for instrumented posturography: a systematic review and meta-analysis. Front Hum Neurosci. 2024;18:1498107. 10.3389/FNHUM.2024.1498107/BIBTEX. 39743990 10.3389/fnhum.2024.1498107 PMC11688309

[15] Lesch KJ, Tuomisto S, Tikkanen HO, Venojärvi M. Validity and reliability of dynamic and functional balance tests in people aged 19–54: A systematic review. Int J Sports Phys Ther. 2024;19:381–93. 10.26603/001C.94612. 38699672 10.26603/001c.94612 PMC11065456

[16] Rodrigues CAS, Albano TR, Pereira Melo AK, Azevedo Tavares ML, Lima PO, de Almeida P. Y-Balance test after ACL reconstruction: the relationship with knee and hip muscle strength, ankle dorsiflexion range of motion and postural stability. J Bodyw Mov Ther. 2024;40:2099–104. 10.1016/J.JBMT.2024.10.016. 39593570 10.1016/j.jbmt.2024.10.016

[17] Almeida GPL, Monteiro IO, Marizeiro DF, Maia LB, de Paula Lima PO. Y balance test has no correlation with the stability index of the biodex balance system. Musculoskelet Sci Pract. 2017;27:1–6. 10.1016/J.MSKSP.2016.11.008. 28637596 10.1016/j.msksp.2016.11.008

[18] Seong Kim J, Jae Hwang U, Young Choi M, Hwan Kong D, Sung Chung K, Ku Ha J, et al. Correlation between Y-Balance test and Balance, functional Performance, and outcome measures in patients following ACL reconstruction. Int J Sports Phys Ther. 2022;17:2022. 10.26603/001c.31873. 10.26603/001c.31873 PMC8805125 35136688

[19] Abde RP, Luciana A, Acde R. Balance performance in sedentary and active healthy young individuals – a cross-sectional study. Phys Educ Students. 2020;24:115–9. 10.15561/20755279.2020.0207.

[20] TURGUT MC, AYAS MS. Comparison of athletes and Non-Athletes on meniscal repair outcomes: A retrospective study. Türkiye Klinikleri Spor Bilimleri Dergisi. 2022;14:56–60. 10.5336/SPORTSCI.2021-85324.

[21] Oleksy Ł, Mika A, Sulowska-Daszyk I, Szymczyk D, Kuchciak M, Stolarczyk A, et al. Standard RTS criteria effectiveness verification using FMS, Y-balance and TJA in footballers following ACL reconstruction and mild lower limb injuries. Sci Rep 2021. 2021;11:1. 10.1038/s41598-021-81152-4. 10.1038/s41598-021-81152-4 PMC7810698 33452381

[22] Bühl L, Müller S, Nüesch C, Pagenstert G, Mündermann A, Egloff C. Functional leg performance 2 years after ACL surgery: a comparison between InternalBraceTM-augmented repair versus reconstruction versus healthy controls. J Orthop Traumatol. 2023;24. 10.1186/S10195-023-00723-5. 10.1186/s10195-023-00723-5 PMC10513977 37735271

[23] Tegner Y, Lysholm J. Rating systems in the evaluation of knee ligament injuries. Clin Orthop Relat Res. 1985;198:43–9. 10.1097/00003086-198509000-00007. 4028566

[24] Roos EM, Roos HP, Lohmander LS, Ekdahl C, Beynnon BD. Knee injury and osteoarthritis outcome score (KOOS)--development of a self-administered outcome measure. J Orthop Sports Phys Ther. 1998;28:88–96. 10.2519/JOSPT.1998.28.2.88. 9699158 10.2519/jospt.1998.28.2.88

[25] Irrgang JJ, Anderson AF, Boland AL, Harner CD, Kurosaka M, Neyret P, et al. Development and validation of the international knee Documentation committee subjective knee form. Am J Sports Med. 2001;29:600–13. 10.1177/03635465010290051301. 11573919 10.1177/03635465010290051301

[26] Bipodal Balance. Procedures & Testing • Kinvent. https://kinvent.com/pdf-for-ebook/bipodal-balance-procedures-testing/. Accessed 1 Jan 2026.

[27] Marcori AJ, Monteiro PHM, Oliveira JA, Doumas M, Teixeira LA. Single leg balance training: A systematic review. Percept Mot Skills. 2022;129:232–52. 10.1177/00315125211070104. 35084234 10.1177/00315125211070104

[28] Plisky GPP, Butler RJ, Kiesel KB, Underwood FB, Elkins B. The reliability of an instrumented device for measuring components of the star excursion balance test. N Am J Sports Phys Ther. 2009;4:92. 21509114 PMC2953327

[29] Clagg S, Paterno MV, Hewett TE, Schmitt LC. Performance on the modified star excursion balance test at the time of return to sport following anterior cruciate ligament reconstruction. J Orthop Sports Phys Ther. 2015;45:444–52. 10.2519/JOSPT.2015.5040. 25899211 10.2519/jospt.2015.5040

[30] Mattacola CG, Perrin DH, Gansneder BM, Gieck JH, Saliba EN, McCue FC. Strength, functional Outcome, and postural stability after anterior cruciate ligament reconstruction. J Athl Train. 2002;37:262. 12937583 PMC164354

[31] Henriksson M, Ledin T, Good L. Postural control after anterior cruciate ligament reconstruction and functional rehabilitation. Am J Sports Med. 2001;29:359–66. 10.1177/03635465010290031801. 11394609 10.1177/03635465010290031801

[32] Lennon AR, Killelea C, Faherty MS, Sell TC. No difference in static postural stability bilaterally or versus control at 12 weeks after anterior cruciate ligament reconstruction. Int J Sports Phys Ther. 2025;20:1684. 10.26603/001C.147061. 41346849 10.26603/001c.147061 PMC12673949

[33] Plisky P, Schwartkopf-Phifer K, Huebner B, Garner MB, Bullock G. Systematic review and Meta-Analysis of the Y-Balance test lower quarter: Reliability, discriminant validity, and predictive validity. Int J Sports Phys Ther. 2021;16:1190. 10.26603/001C.27634. 34631241 10.26603/001c.27634 PMC8486397

[34] Petersen W, Taheri P, Forkel P, Zantop T. Return to play following ACL reconstruction: a systematic review about strength deficits. Arch Orthop Trauma Surg. 2014;134:1417–28. 10.1007/S00402-014-1992-X/TABLES/4. 25091127 10.1007/s00402-014-1992-x

[35] Kaur N, Bhanot K, Ferreira G. Lower extremity and trunk electromyographic muscle activity during performance of the Y-Balance test on stable and unstable surfaces. Int J Sports Phys Ther. 2022;17:483–92. 10.26603/001C.32593. 35391869 10.26603/001c.32593 PMC8975559

[36] Kang MH, Kim GM, Kwon OY, Weon JH, Oh JS, An DH. Relationship between the kinematics of the trunk and lower extremity and performance on the Y-Balance test. PM&R. 2015;7:1152–8. 10.1016/J.PMRJ.2015.05.004. 25978949 10.1016/j.pmrj.2015.05.004

[37] Smith CA, Chimera NJ, Warren M. Association of Y balance test reach asymmetry and injury in division I athletes. Med Sci Sports Exerc. 2015;47:136–41. 10.1249/MSS.0000000000000380. 24870573 10.1249/MSS.0000000000000380

[38] Gonell AC, Romero JAP, Soler LM. Relationship between they balance test, scores and soft tissue injury incidence in a soccer team. Int J Sports Phys Ther. 2015;10:955. 26673848 PMC4675196

[39] Ruffe NJ, Sorce SR, Rosenthal MD, Rauh MJ. Lower quarter- and upper quarter y balance tests as predıctors of runnıng-related ınjurıes ın hıgh school cross-country runners. Int J Sports Phys Ther. 2019;14:695. 10.26603/ijspt20190695. 31598407 PMC6769269

[40] Bartels T, Brehme K, Pyschik M, Pollak R, Schaffrath N, Schulze S, et al. Postural stability and regulation before and after anterior cruciate ligament reconstruction - A two years longitudinal study. Phys Ther Sport. 2019;38:49–58. 10.1016/J.PTSP.2019.04.009. 31051428 10.1016/j.ptsp.2019.04.009

[41] Kim J-G, Lee D-W, Bae K-C, Choi B-C, Yang S-J, Cho S-I et al. Correlation of Y balance with clinical scores and functional tests after anterior cruciate ligament reconstruction in young and Middle-Aged patients. 2022. 10.4055/cios21131. 10.4055/cios21131 PMC9880508 36778986

[42] Kim JG, Lee DW, Bae KC, Choi BC, Yang SJ, Cho SI, et al. Correlation of Y balance with clinical scores and functional tests after anterior cruciate ligament reconstruction in young and Middle-Aged patients. Clin Orthop Surg. 2023;15:50–8. 10.4055/CIOS21131. 36778986 10.4055/cios21131 PMC9880508

[43] Howells BE, Ardern CL, Webster KE. Is postural control restored following anterior cruciate ligament reconstruction? A systematic review. Knee Surg Sports Traumatol Arthrosc. 2011;19:1168–77. 10.1007/S00167-011-1444-X/METRICS. 21344230 10.1007/s00167-011-1444-x

